# Long noncoding RNA RHPN1-AS1 promotes colorectal cancer progression via targeting miR-7-5p/OGT axis

**DOI:** 10.1186/s12935-020-1110-9

**Published:** 2020-02-18

**Authors:** Wei Zheng, Hui Li, Hui Zhang, Chao Zhang, Zhonglin Zhu, Hong Liang, Yifeng Zhou

**Affiliations:** 1 Department of Gastrointestinal Surgery, Henan Provincial People’s Hospital, Zhengzhou University People’s Hospital, Zhengzhou, 450003 Henan China; 2 Department of Gastroenterology, Affiliated Hangzhou First People’s Hospital, Zhejiang University School of Medicine, No. 261 Huanshan Road, Hangzhou, 310006 Zhejiang China

**Keywords:** RHPN1-AS1, miR-7-5p, OGT, Colorectal cancer

## Abstract

**Background:**

Rhophilin Rho GTPase binding protein 1 antisense RNA 1 (RHPN1-AS1) is a newly discovered oncogene in several diseases, such as breast cancer, non-small cell lung cancer and uveal melanoma. Nevertheless, its molecular role in colorectal cancer (CRC) remains unknown. This paper explored the role of RHPN1-AS1 in CRC progression.

**Methods:**

qRT-PCR was used to detect relevant RNAs expression. CCK-8, EdU, flow cytometry, Transwell and western blot assays were performed to investigate the function of RHPN1-AS1 in CRC cells. Xenograft model was constructed to evaluate the effects of RHPN1-AS1 on tumor growth in vivo. Mechanical experiments were performed to investigate the relationship between relative genes.

**Results:**

RHPN1-AS1 was significantly overexpressed in CRC cell lines. Knockdown of RHPN1-AS1 could inhibit cell proliferation, while stimulating cell apoptosis in vitro. Cell migration and invasion abilities were greatly suppressed after silencing RHPN1-AS1. Besides, signal transducer and activator of transcription 3 (STAT3) served as transcription factor of RHPN1-AS1. Moreover, miR-7-5p was identified as a target of RHPN1-AS1 and was negatively regulated by RHPN1-AS1 in CRC. MiR-7-5p inhibition rescued the oncogenic function of RHPN1-AS1. Additionally, *O*-GlcNAcylation transferase (OGT) was the downstream target of miR-7-5p. OGT overexpression could abrogate the anti-tumor effects of RHPN1-AS1 knockdown on CRC.

**Conclusion:**

RHPN1-AS1 regulates CRC by mediating OGT through sponging miR-7-5p, suggesting that RHPN1-AS1 might be a potential therapeutic target for CRC.

## Background

On a global scale, colorectal cancer (CRC) is one of the most common malignant [1]. Annually, approximately 1.2,000,000 new cases were diagnosed and 60,000 death cases happened [2]. The overall occurrence and mortality are declining due to the advance in new techniques and therapeutic methods for CRC predication and treatment [3]. However, the overall 5-year survival rate is still lower than anticipation as metastatic CRC frequently occurred in patients at the time of diagnosis [4, 5]. Hence, it is paramount to reveal the underlying mechanism behind the progression of CRC, so as to identify potential novel diagnostic and prognostic biomarkers.

The newly-identified non-coding RNAs (ncRNAs) are a cluster of RNAs with the ability of transcriptional control and post-transcriptional mediation, but they lack the potential to encode proteins [5–8]. Long non-coding RNAs (lncRNAs) are a kind of ncRNAs containing over 200 nucleotides in length. They have been extensively studied in the carcinogenesis of many diseases, including cancer. Moreover, recent findings manifested that lncRNAs are involved in a series of biological processes, such as cell proliferation, apoptosis, invasion and migration [9, 10]. Furthermore, lncRNAs have been identified as new biomarkers of many malignancies, since they are associated with the complicated pathogenesis of malignancies [11, 12]. In addition, the prevalent competing endogenous RNA (ceRNA) mechanism has revealed the crucial regulatory role of lncRNAs on downstream RNAs, consequently impacting various physiological and pathophysiological activities within cells [13]. To date, the pathologic roles of most lncRNAs remain undiscovered, which indicates the extensive application potential of lncRNAs in the prediction and treatment of different cancers.

In CRC, the expression status and functional role of some lncRNAs were investigated. For instance, lncRNA XIRP2-AS1 was discovered significantly lowly expressed in CRC tissues and could serve as a favorable biomarker for CRC patients [14]. Silencing lncRNA AWPPH significantly curbed CRC cell proliferation via down-regulating the expression of GLUT-1 [15]. FEZF1-AS1 has been reported to accelerate the progression of CRC via up-regulating the expression of NT5E through sponging miR-30a-5p [16].

RHPN1-AS1, as a newly identified lncRNA, has been revealed to promote the carcinogenesis of head and neck squamous cell carcinoma [17]. Furthermore, it has been reported as a potential clinical biomarker and participated in important biological processes and pathways in non-small cell lung cancer (NSCLC) [18]. Nevertheless, the molecular role of RHPN1-AS1 behind the carcinogenesis and development of CRC has not been explored yet. Therefore, the aim of present study is to explore the cellular function of RHPN1-AS1 in CRC, which might contribute to providing some novel thoughts about finding effective treatment targets for CRC patients.

## Materials and methods

### Cell culture

Normal human colorectal mucosal cell (FHC) and CRC cells (SW620, SW480, HCT-116, HT29) were bought from Chinese Academy of Sciences (Beijing, China). Human kidney cell (293T) was gained from Genechem (Shanghai China). Cells were grown in RPMI-1640 medium (Invitrogen, Carlsbad, CA, USA) adding 10% fetal bovine serum (FBS; Invitrogen), 1% penicillin/streptomycin (Sigma-Aldrich, Milan, Italy) and cultured in a 5% CO_2_ incubator at 37 °C.

### Cell transfection

HCT-116 and HT29 cells were transfected with specific shRNAs against RHPN1-AS1 (sh-RHPN1-AS1#1/#2), STAT3 (sh-STAT3), OGT (sh-OGT#1/#2) and their corresponding control group (sh-Ctrl), and pcDNA3.1/STAT3, pcDNA3.1/OGT and the empty pcDNA3.1 vector, respectively. The miR-7-5p inhibitor, miR-7-5p mimics, NC mimics and NC inhibitor were synthesized by GenePharma (Shanghai, China). Lipofectamine 2000 (Invitrogen) was used for executing transfection experiments for 48 h.

### Quantitative real time polymerase chain reaction (qRT-PCR)

TRIzol reagent (Invitrogen) was employed to obtain total RNA from cells. Reverse Transcription Kit (Applied Biosystems, CA, USA) or Taqman Advanced miRNA cDNA Synthesis Kit (Thermo Fisher Scientic) was used to reverse transcribe total RNA into cDNA. qRT-PCR was performed on Bio-Rad CFX96 System utilizing the ABI StepOnePlus system (Applied Biosystems). Fold expression changes were calculated with 2^−∆∆Ct^ method, and GAPDH/U6 was endogenous control.

### Cell counting kit-8 (CCK-8) assay

1 × 10^3^ cells were added to 96-well plates with culture media. The medium of every well was replaced by a fresh culture media containing 10 µL CCK8 at specific time points. The cells were cultivated for additional 4 h. The absorbance at 450 nm was measured via a microplate reader (Olympus, Tokyo, Japan).

### EdU incorporation assay

EdU (5-ethynyl-2′-deoxyuridine) assay was performed to detect cell proliferation ability using the Click-iT EdU Imaging Kit (Invitrogen) in line with manufacturer’s directions. Transfected HT29 and HCT-116 were maintained into 96-well plate. After incubation of 48 h, 100 μl medium with 50 μM EdU was added into all wells. Cells underwent 2 h incubation at 37 °C and were fixed with 4% paraformaldehyde. Nuclei were dyed in DAPI for 30 min. EdU-positive cells were visualized using a microscope (Olympus). Proliferation rate was determined by calculating the ratio of the fluorescent positive cells to total cells.

### Flow cytometry analysis

HCT-116 and HT29 cells were reaped and cultured in 6-well plates after transfection. Followed by rinsing with PBS, Annexin V-FITC and Propidium iodide (PI) were used for double staining. Apoptosis rate was measured via a flow cytometry (BD Biosciences, Beijing, China).

### Transwell assay

HCT-116 and HT29 cells were seeded into the upper chamber (Corning, NY, USA) with or without pre-coated Matrigel (Becton–Dickinson, San Jose, CA, USA) for invasion or migration analysis. 5 × 10^4^ cells with serum-free medium were added into the top compartment, while medium containing 10% FBS was placed into the bottom compartment later. After incubation for 24 h (migration) or 48 h (invasion), cells on the underside of membrane were immobilized and dyed in methanol or crystal violet. Finally, cells were counted in 5 random chosen fields under the microscope (Olympus).

### Western blot

Total proteins were extracted with RIPA lysis buffer (Invitrogen). Protein concentrations were examined by BCA kit. There was 10% SDS-PAGE for the isolation of proteins. And then proteins were moved on PVDF membranes. After being blocked with PVDF, membranes were cultured with primary antibodies for E-cadherin (ab194982), N-cadherin (ab202030), Vimentin (ab193555), Slug (ab51772), Twist (ab187008), OGT (ab96718) and GAPDH (ab8245) from Abcam (Cambridge, CA, USA). Then, secondary antibodies were added for 1 h cultivation. The amount of protein was determined using chemiluminescence detection system.

### Subcellular fractionation

Both nuclear and cytoplasmic RNA from CRC cells were separated with PARIS Kit (Ambion, Austin, TX, USA). Subsequently, qRT-PCR was employed for detecting the relative expression level of RHPN1-AS1. GAPDH/U6 was used as cytoplasmic/nuclear control.

### Fluorescence in situ hybridization (FISH) assay

RHPN1-AS1 FISH probe was constructed by Ribo Bio Technology (Guangzhou, China). Cells were fixed in 4% paraformaldehyde, and then permeabilized in PBS which was mixed with 0.5% Triton X-100. After that, cells were hybridized with Cy3 labeled RNA FISH probe at 37 °C. Subsequently, cells were stained by the use of Hoechst. The presence of RHPN1-AS1 was visualized using confocal microscopy (Olympus).

### Luciferase reporter assay

The wild-type (WT) and mutant (MUT) binding sites of miR-7-5p in RHPN1-AS1 sequence or OGT 3′-UTR was sub-cloned into pmirGLO dual-luciferase vector to construct RHPN1-AS1-WT/MUT, OGT-WT/MUT, and then co-transfected with miR-7-5p mimics or NC mimics into HCT-116, HT29 and HEK-293T cells, respectively. The pGL3-RHPN1-AS1 promoter was co-transfected with pcDNA3.1/STAT3, pcDNA3.1, sh-STAT3 or sh-Ctrl into HEK-293T cell. Dual-Luciferase Reporter Assay System (Promega, USA) detected the luciferase activity.

### RNA pull down assay

The RHPN1-AS1 WT, RHPN1-AS1 MUT or NC was biotin labeled into Biotin-RHPN1-AS1 WT, Biotin-RHPN1-AS1 MUT or Bio-NC, severally. Afterwards, cell lysates were incubated with biotinylated RNA overnight and M-280 streptavidin magnetic beads (Sigma-Aldrich) were added for cultivation for 48 h. Relative enrichment of miR-7-5p was assayed by RT-qPCR.

### Chromatin immunoprecipitation (ChIP) assay

Cells were subjected to chromatin immunoprecipitation based upon an EZ-ChIP chromatin immunoprecipitation kit (Millipore, Bedford, MA, USA). Briefly, cells were cross-linked by formaldehyde. Cell lysates were gathered and sonicated to shear DNA. After that, chromatin was immunoprecipitated using anti-STAT3 (Millipore), while IgG was the negative control. The relative enrichment of RHPN1-AS1 was evaluated by qRT-PCR.

### RNA immunoprecipitation (RIP) assay

EZ-Magna RIP kit (Millipore) was applied for conducting RIP assays. HCT-116 and HT29 cells were lysed in RIP lysis buffer, and incubated with anti-Ago2 (Millipore) and IgG (Millpore). The relative expression levels of miR-7-5p, RHPN1-AS1 and OGT were examined using qRT-PCR analysis.

### Tumor xenograft experiment

Female nude mice were gained from Shi Laike Company (Shanghai, China). Cells infected with sh-RHPN1-AS1#1 or sh-Ctrl was injected subcutaneously into the hip back of mice. 4 days later, tumor growth was recorded by measuring their volume. After 4 weeks, nude mice were euthanized to weigh the tumors. This study was progressed with the approval of the Ethics Committee of Henan Provincial People’s Hospital.

### Statistical analysis

Experimental data were manifested as mean ± SD. All statistical analyses were achieved using the GraphPad software 6.0 (GraphPad Inc., San Diego, CA, USA). Each experiment was done at least three times. The variance among groups was analyzed via Student’s t-test or one-way ANOVA. Differences were seen as statistically significant when P < 0.05.

## Results

### RHPN1-AS1 is aberrantly up-regulated in CRC and knockdown of RHPN1-AS1 suppresses the malignant behaviors of CRC

**Fig. 1:**
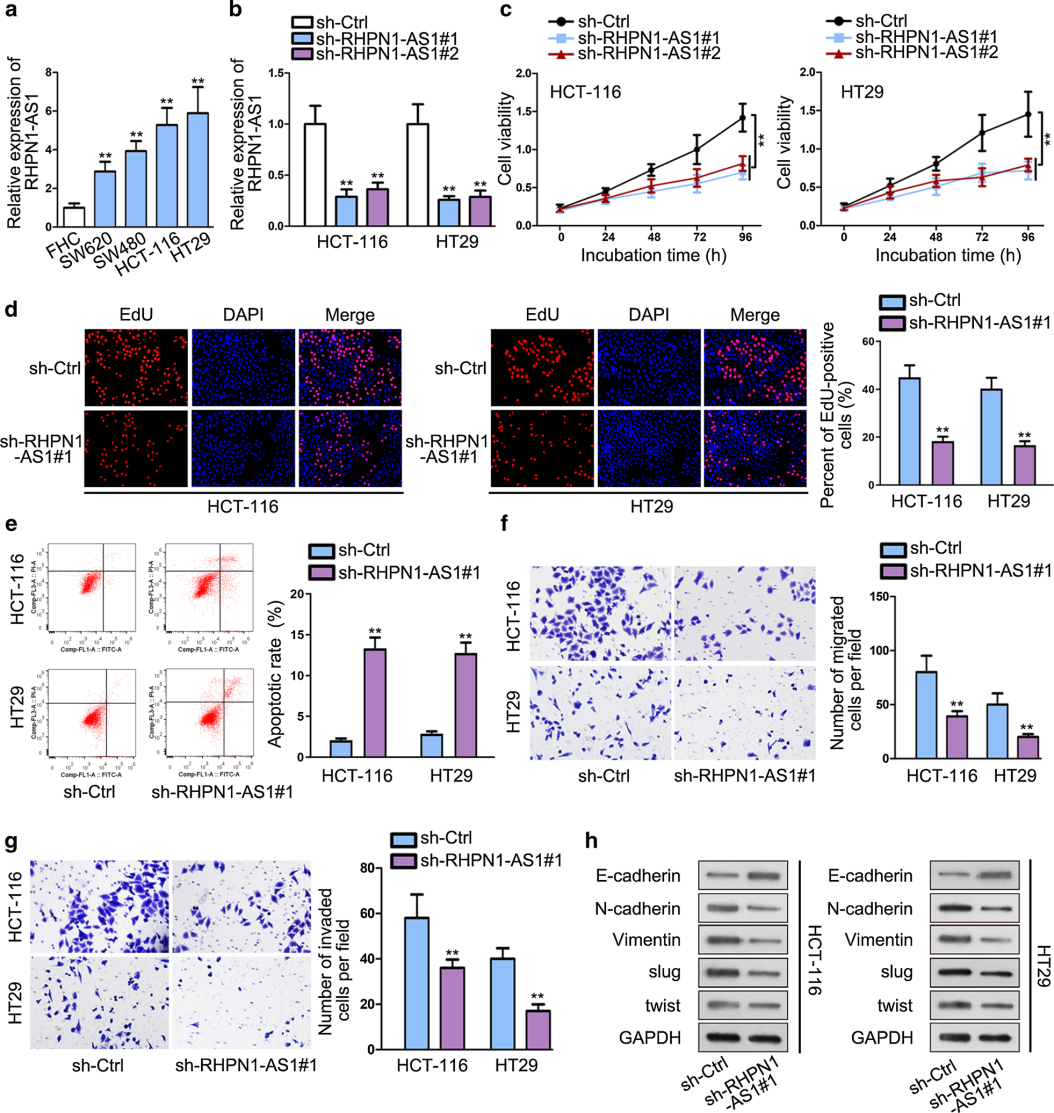
RHPN1-AS1 is aberrantly up-regulated in CRC and knockdown of RHPN1-AS1 suppresses the malignant behaviors of CRC. **a** qRT-PCR assay was used to examine RHPN1-AS1 expression in CRC cell lines and non-tumor cell line. **b** The transfection efficiency of sh-RHPN1-AS1#1/#2 plasmid was detected by qRT-PCR. **c**, **d** CCK-8 and EdU were performed to investigate cell proliferation. **e** Flow cytometry was used to determine apoptosis. **f**, **g** Transwell assays were conducted to evaluate cell migration and invasion abilities respectively. **h** Western blot was performed to examine EMT-related protein expression. ^**^P < 0.01

To determine the expression of RHPN1-AS1 in CRC, we performed qRT-PCR analysis. We found that RHPN1-AS1 was predominantly up-regulated in CRC cell lines (SW620, SW480, HCT-116 and HT29) than healthy colon cell line (FHC). HCT-116 and HT29 cell lines contained the most obviously high expression of RHPN1-AS1 and were chose to apply in the following assays (Fig. 1a). Subsequently, loss-of-function assay was adopted to investigate the role of RHPN1-AS1 in CRC cells. qRT-PCR result manifested that the inhibiting effect of sh-RHPN1-AS1#1 on RHPN1-AS1 in HCT-116 and HT29 cell lines was more visually than sh-RHPN1-AS1#2. Accordingly, sh-RHPN1-AS1#1 was used in next researches (Fig. 1b). Then, CCK-8 and EdU assays were performed to determine cell proliferation after knocking down RHPN1-AS1. We observed that silencing RHPN1-AS1 significantly detered cell proliferation than control group (Fig. 1c, d). Flow cytometry assay was conducted to assess cell apoptosis ability. We found a significant increase in the fraction of apoptosis cells after silencing RHPN1-AS1, which indicated that knockdown of RHPN1-AS1 promoted CRC cell apoptosis (Fig. 1e). Besides, transwell assays showed that knockdown of RHPN1-AS1 diminished migrated and invaded cells compared with control group (Fig. 1f, g). (Epithelial-mesenchymal transition) EMT is a crucial phase in CRC metastasis process [19]. Western blot assay was conducted to investigate the expression status of EMT-related proteins. We found an increase of E-cadherin, while a reduction of N-cadherin, Vimentin, slug and twist after RHPN1-AS1 knockdown (Fig. 1h). This result further proved the anti-migration and anti-invasion effects of silencing RHPN1-AS1 on CRC cells. Above all, RHPN1-AS1 is aberrantly up-regulated in CRC and knockdown of RHPN1-AS1 suppresses the malignant behaviors of CRC.

### STAT3 stimulates the transcription of RHPN1-AS1

**Fig. 2:**
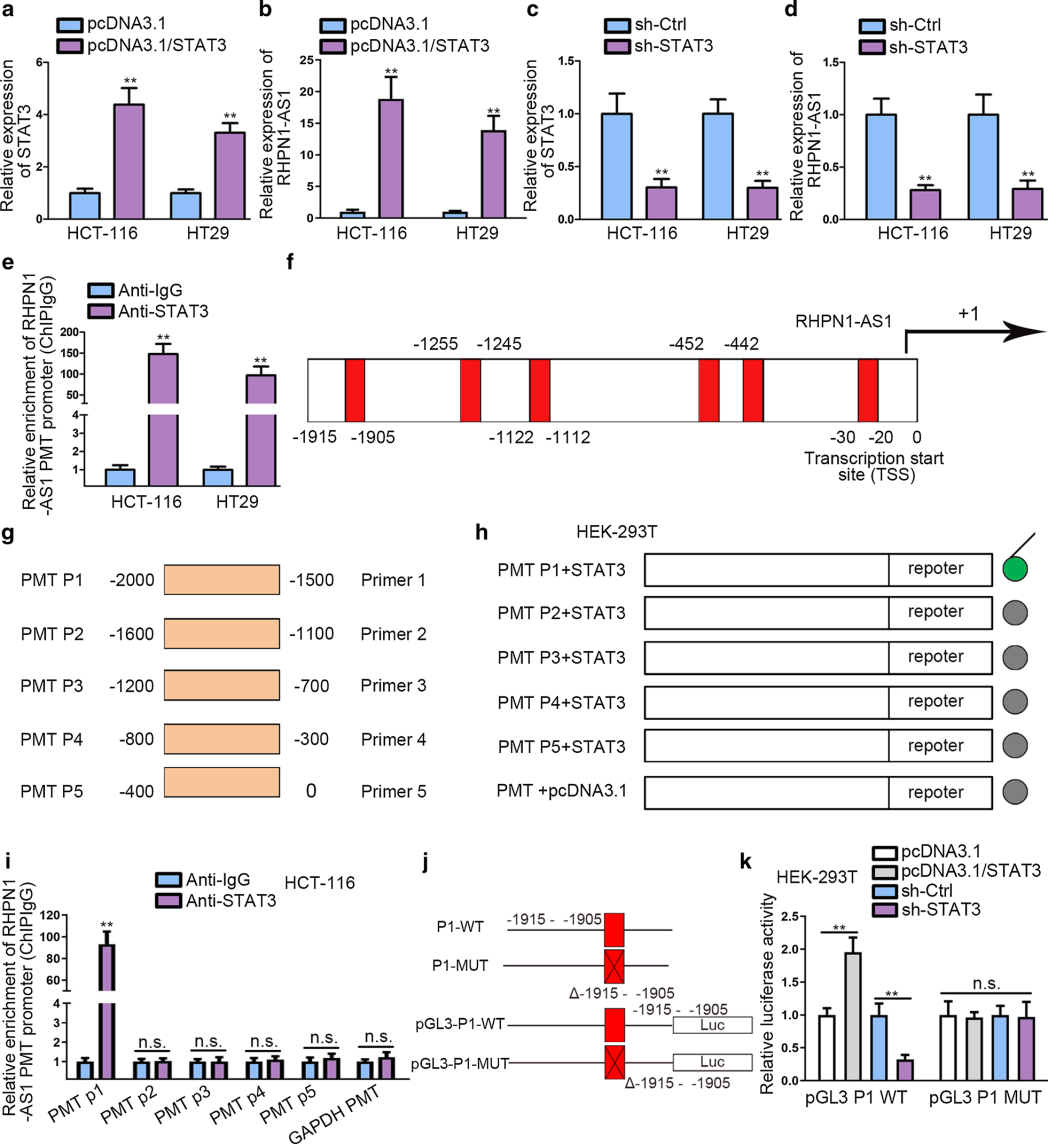
STAT3 stimulates the transcription of RHPN1-AS1. **a** qRT-PCR analysis was performed to detect the expression of STAT3 after transfecting with pcDNA3.1 and pcDNA3.1/STAT3 plasmids. **b** qRT-PCR was performed to study the effects of overexpressing STAT3 on RHPN1-AS1. **c** qRT-PCR was conducted to determine the expression of STAT3 after knockdown it. **d** qRT-PCR was performed to study the effects of silencing STAT3 on RHPN1-AS1. **e** ChIP assay using antibody targeting STAT3 and IgG was performed to investigate the binding relation of STAT3 and RHPN1-AS1 promoter. **f** Predicted binding site of STAT3 on RHPN1-AS1 promoter region was obtained by Jaspar database. **g** RHPN1-AS1 promoter region was divided into five truncations according to the predicted binding site. **h** Luciferase reporter assay was performed to study which truncation in RHPN1-AS1 promoter region interacted with STAT3 in HEK-293T cell line. **i** ChIP analysis of the binding relation between RHPN1-AS1 promoter and STAT3 (GAPDH PMT served as negative control). **j** The wild type and mutant type of potential binding site in RHPN1-AS1 PMT P1 were constructed and sub-cloned into pGL3 luciferase vector. **k** HEK-293T was transfected with sh-Ctrl, sh-STAT3, pcDNA3.1 and pcDNA3.1/STAT3 vector and a luciferase reporter for 48 h was performed. ns meant no significance. ^**^P < 0.01

Accumulating evidences manifested that some epigenetic regulators and key transcription factors contributed to lncRNAs dysregulation in human cancers [20, 21]. We found that the transcription factor STAT3 might associate with RHPN1-AS1 promoter region on UCSC (http://genome.ucsc.edu/) and Jaspar (http://jaspar.genereg.net/) databases. STAT3 has been revealed to function as oncogene in CRC [22]. Additionally, it has been found to engage in the activation of IL-6/STAT3 pathway in CRC [23]. To investigate the effects of STAT3 on RHPN1-AS1, the overexpression efficiency of STAT3 was measured in the first place (Fig. 2a). Then qRT-PCR result manifested that the expression of RHPN1-AS1 was increased after transfecting pcDNA3.1/STAT3 plasmids into CRC cells (Fig. 2b). Afterwards, we knocked down STAT3 and the expression of STAT3 declined greatly (Fig. 2c). Besides, we noticed a significant down-regulation of RHPN1-AS1 after STAT3 knockdown (Fig. 2d). These results indicated that STAT3 could play a crucial role in regulating the expression of RHPN1-AS1. ChIP assay was performed to determine the binding relation between STAT3 and RHPN1-AS1 promoter (PMT) using anti-IgG and anti-STAT3. The results manifested that RHPN1-AS1 PMT was significantly enriched in anti-STAT3 group than anti-IgG group, which indicated that STAT3 could bind to RHPN1-AS1 PMT region (Fig. 2e). Additionally, the potential binging sites of STAT3 in RHPN1-AS1 PMT promoter area were obtained by utilizing UCSC and Jaspar (Fig. 2f). To determine the specific binding segments, we divided RHPN1-AS1 PMT into five segments according to the putative binding sites (Fig. 2g). Furthermore, luciferase reporter assay was performed to investigate the promoter activity after the five truncations of pGL3-RHPN1-AS1 promoter were co-transfected with pcDNA3.1 or pcDNA3.1/STAT3 in HEK-293T cells. We observed that STAT3 overexpression could significantly enhance the transcription activity of RHPN1-AS1 after co-transfecting with pGL3-RHPN1-AS1 PMT P1, as illustrated in Fig. 2h. Furthermore, we noticed that STAT3 could bind to P1 truncation from ChIP data (Fig. 2i). These findings revealed that STAT3 bound to RHPN1-AS1 promoter at about − 2000 to − 1500 bp upstream transcription start site (TSS). To determine whether STAT3 bound to RHPN1-AS1 promoter at the putative binding site existing in P1, we constructed P1-WT (-1915 to - 1905) and P1-MUT (Δ-1915 to -1905) and transfected them into pGL3 vector respectively (Fig. 2j). We observed that the promoter activity of P1-WT was significantly enhanced after co-transfecting with pcDNA3.1/STAT3, but weakened in sh-STAT3 group in HEK-293T. Conversely, P1-MUT (Δ-1915 to -1905), which lacked -1915 to -1905 sites, showed barely no promoter activity change (Fig. 2k). To sum up, these results demonstrated that STAT3 plays a positive regulatory role in the transcription of RHPN1-AS1 via binding to -1915 to -1905 site upstream TSS in RHPN1-AS1 promoter region.

### RHPN1-AS1 directly interacts with miR-7-5p and inhibits miR-7-5p expression

**Fig. 3:**
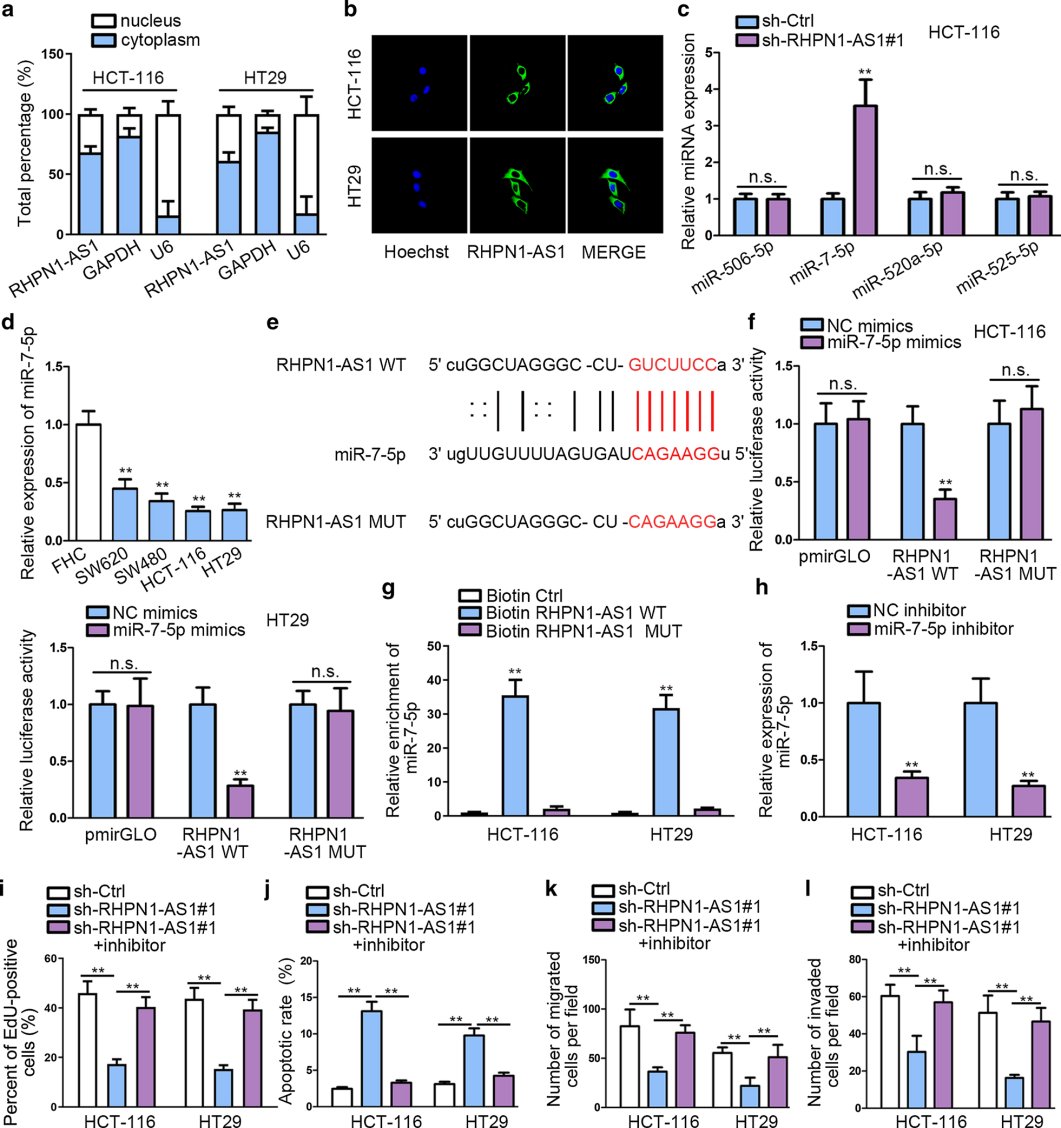
RHPN1-AS1 directly interacts with miR-7-5p and inhibits miR-7-5p expression. **a**, **b** Subcellular fractionation and FISH assays were performed to detect the subcellular location of RHPN1-AS1. **c** qRT-PCR analysis was performed to evaluate miRNA expression after silencing RHPN1-AS1. **d** qRT-PCR analysis was conducted to investigate the expression status of miR-7-5p in CRC cell lines and normal cell lines. **e** Putative and mutated miR-7-5p binding site in the sequence of RHPN1-AS1 was shown by utilizing Starbase. **f** Dual luciferase reporter assays were performed to study the interaction between miR-7-5p and RHPN1-AS1. **g** RNA pull down was performed to investigate the association between miR-7-5p and RHPN1-AS1. **h** The inhibition efficiency of miR-7-5p inhibitor was examined by qRT-PCR. **i** EdU was performed to detect cell proliferation in sh-Ctrl, sh-RHPN1-AS1#1 or sh-RHPN1-AS1#1 + inhibitor transfected groups. **j** Flow cytometry was conducted to investigate apoptosis of cells transfected by sh-Ctrl, sh-RHPN1-AS1#1 or sh-RHPN1-AS1#1 + inhibitor. **k**, **l** Transwell assays were used to evaluate migration and invasion of cells transfected by sh-Ctrl, sh-RHPN1-AS1#1 or sh-RHPN1-AS1#1 + inhibitor. ns meant no significance. ^**^P < 0.01

We determined the location of RHPN1-AS1 via subcellular fractionation assay and FISH analysis. Both results indicated that RHPN1-AS1 was largely situated in cytoplasm (Fig. 3a, b). We screened out the combinable miRNA via Starbase database (http://starbase.sysu.edu.cn/) according to several restricted binding conditions (class ≥ 7 mer-m8; Ago ExpNum ≥ 1, target gene: RHPN1-AS1). And only four miRNAs were qualified. Subsequently, qRT-PCR was performed to study the effects on these miRNA after silencing RHPN1-AS1. The expression of miR-7-5p increased most significantly after knockdown RHPN1-AS1 compared with control group, while other miRNAs showed no significant up-regulation (Fig. 3c). Besides, miR-7-5p has been validated to be down-regulated in CRC, and served as a tumor suppressor by targeting oncogene KLF4 in CRC [24]. Therefore, we chose miR-7-5p as candidate miRNA for present study. We identified a significant down-regulation of miR-7-5p in CRC cell lines compared with normal colon cell line (Fig. 3d). Putative binding site of miR-7-5p in the sequence of RHPN1-AS1 was predicted and mutated accordingly, as shown on Fig. 3e. Next, dual luciferase reporter assays were performed in HCT-116 and HT19 to examine the physical association between RHPN1-AS1 and miR-7-5p. We observed that miR-7-5p mimics transfection evidently attenuated the luciferase activity of RHPN1-AS1-WT, but didn’t significantly change that of RHPN1-AS1-MUT (Fig. 3f). qRT-PCR based on RNA pull down findings demonstrated a significant enrichment of miR-7-5p by biotinylated RHPN1-AS1-WT in contrast to negative control groups (Fig. 3g), which further proved that miR-7-5p was the downstream target of RHPN1-AS1.

To determine the influence of miR-7-5p in CRC, we performed rescue experiments after knocking down it (Fig. 3h). EdU result showed that miR-7-5p inhibition could greatly abolish the anti-proliferation role of sh-RHPN1-AS1 in cell proliferation (Fig. 3i). Flow cytometry results demonstrated that miR-7-5p inhibitor could reverse the promoting effects of silencing RHPN1-AS1 on cell apoptosis (Fig. 3j). Transwell assays manifested that miR-7-5p inhibitor could recover CRC migration and invasion abilities repressed by silencing RHPN1-AS1 (Fig. 3k, l). Based on these results, we identify that RHPN1-AS1 plays an oncogenic role in CRC via sponging miR-7-5p.

### OGT is the potential direct target gene of miR-7-5p

**Fig. 4:**
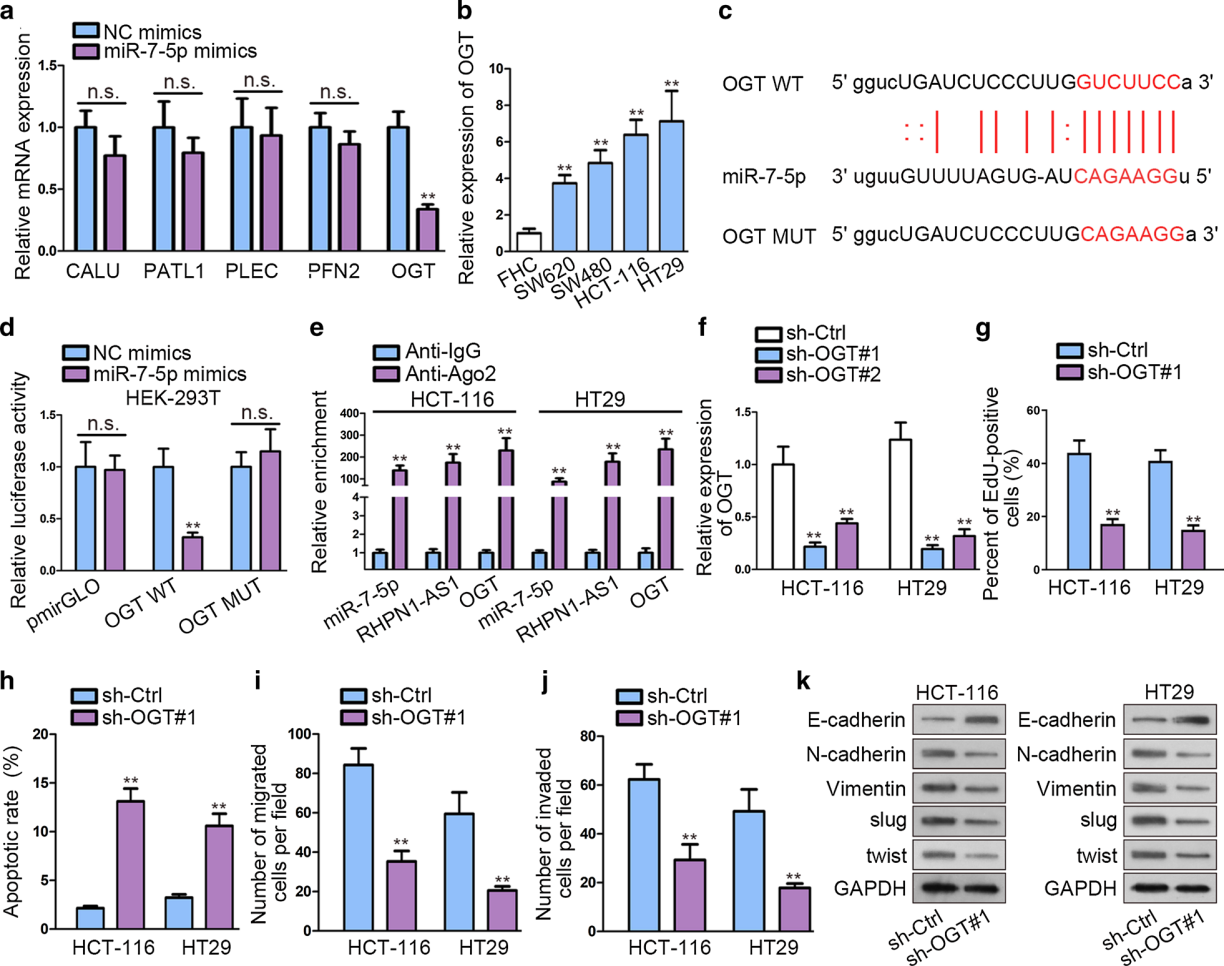
MiR-7-5p targets OGT in CRC. **a** qRT-PCR was performed to study target gene expression change after overexpressing miR-7-5p. **b** qRT-PCR analysis was conducted to detect OGT expression in CRC cell lines and normal healthy cell lines. **c** the binding site between miR-7-5p and OGT was predicted by Starbase, and the corresponding mutated binding site was shown. **d** Luciferase reporter was performed to detect the association between OGT and miR-7-5p in HEK-293T. **e** RIP assay was conducted to determine the physical interaction among miR-7-5p and RHPN1-AS1 and OGT. **f** The inhibiting efficiency of sh-OGT plasmid was investigated by qRT-PCR. **g** EdU was performed to evaluate cell proliferation under sh-Ctrl or sh-OGT#1 transfection condition. **h** Flow cytometry was conducted to examine cell apoptosis when knocking down OGT and sh-Ctrl was control group. **i**, **j**. Transwell assays were used to detect cell migration and invasion in response to silencing OGT. **k** Western blot was performed to measure the expression of EMT-related proteins in sh-Ctrl or sh-OGT#1 transfected cells. ns meant no significance. ^**^P < 0.01

We found five downstream targets of miR-7-5p on Starbase database after strictly limiting binding conditions (Clip data:high stringency ≥ 3; Degradome data: medium stringency ≥ 2; Program Number: 1; Predicted program: microT, miRanda, miRmap, PITA and PicTar; Ago ExpNum ≥ 12). qRT-PCR was performed to detect the effects of transfecting miR-7-5p mimics on mRNAs. OGT expression decreased dramatically after overexpressing miR-7-5p compared with control group (Fig. 4a). Besides, OGT was discovered to be an early biomarker for poor prognosis in esophageal squamous cell carcinoma (ESCC) [25]. In this study, OGT was also aberrantly up-regulated in CRC cell lines (Fig. 4b), which was contrary to the expression profile of miR-7-5p in CRC cell lines. We found the putative miR-7-5p binding site in the 3′ UTR of OGT by utilizing Starbase and mutated the binding site accordingly, as shown on Fig. 4c. Luciferase reporter assay performed on HEK-293T showed that miR-7-5p mimics could dramatically impair the luciferase activity of OGT-WT (Fig. 4d). RIP assay was performed to further ensure this interaction. The result showed that miR-7-5p, OGT and RHPN1-AS1 were significant enriched in anti-Ago2 group compared with IgG control group (Fig. 4e).

Next, loss-of-function experiments were performed to explore the role of OGT in CRC. We noticed that shOGT#1 exhibited better inhibiting effect on OGT by qRT-PCR analysis (Fig. 4f). EdU results demonstrated that cell proliferation was dampened after transfecting sh-OGT#1 in CRC cells (Fig. 4g). Also, flow cytometry showed that silencing OGT could accelerate CRC cell apoptosis (Fig. 4h, Additional file 1: Fig. S1A). In addition, knockdown OGT exerted negative effects on CRC migration and invasion, as shown on Fig. 4i, j. Western blot detected the expression level of EMT-related proteins, revealing that OGT knockdown suppressed EMT process. The level of N-cadherin, Vimentin, slug and twist was decreased, yet that of E-cadherin was increased (Fig. 4k). Collectively speaking, these experiment findings demonstrate that OGT plays an oncogenic role in CRC.

### OGT overexpression abolishes the tumor suppressor effects of RHPN1-AS1 knockdown

**Fig. 5:**
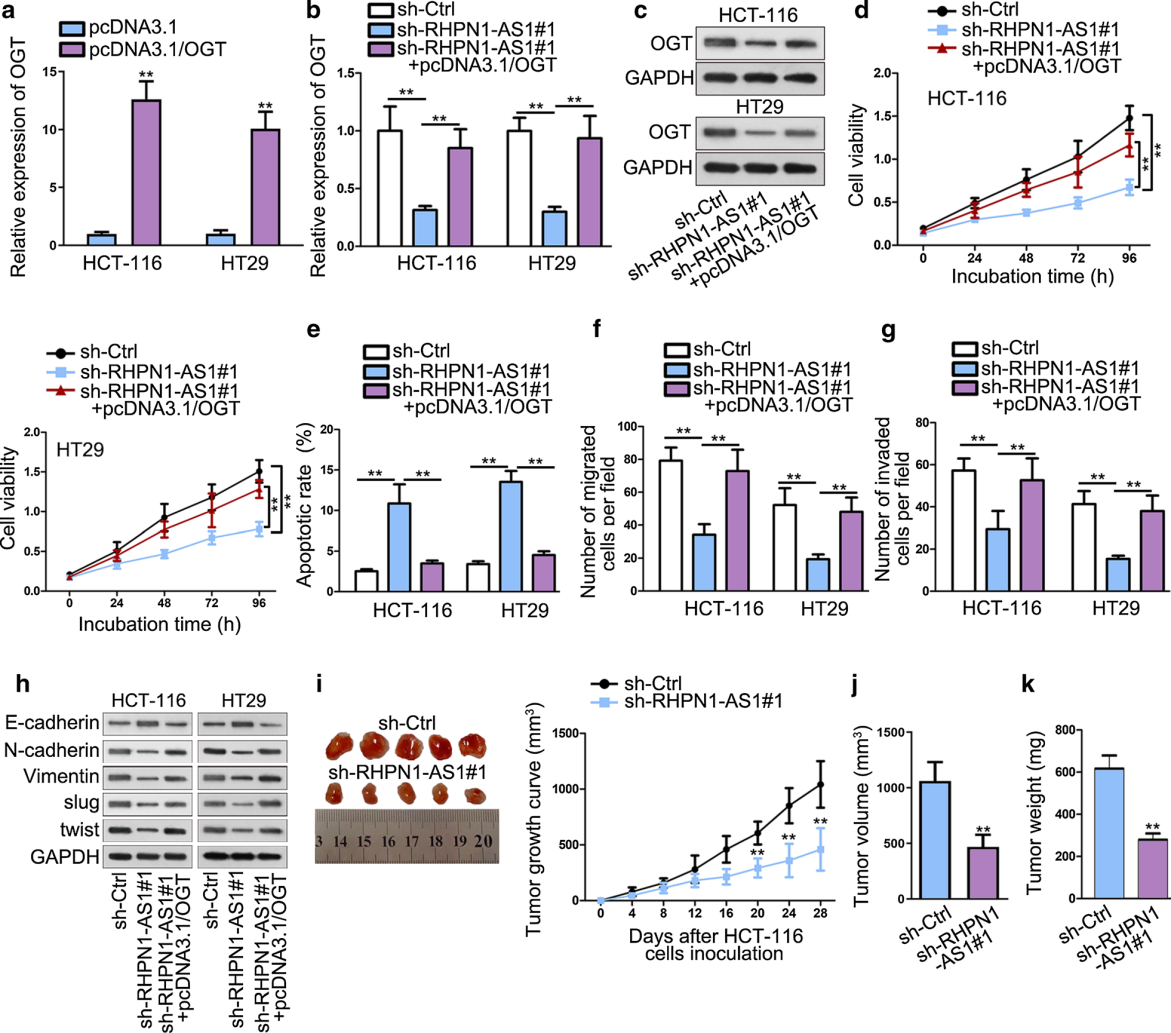
OGT overexpression rescues the oncogenic function of RHPN1-AS1. **a** qRT-PCR analysis was used to determine the expression of OGT after pcDNA3.1 and pcDNA3.1/OGT plasmid transfection. **b**, **c** qRT-PCR and western blot were performed to determine the OGT mRNA and protein expression. **d** CCK-8 assay was performed to evaluate cell vitality. **e** TUNEL assay was conducted to examine cell apoptosis capacity. **f**, **g** Transwell assays were performed to investigate cell migration and invasion abilities respectively. **h** Western blot was performed to measure EMT-related proteins expression change. **i** The photos of tumor in different transfection groups were taken. Xeograft tumor growth curve was plotted after four-week inoculation of HCT-116 cells transfected with sh-RHPN1-AS1#1. **J**, **k** Xeograft tumor volume and weight were measured in sh-Ctrl and sh-RHPN1-AS1 transfected cells in vivo. ^**^P < 0.01

To determine whether RHPN1-AS1 regulated CRC development by sponging miR-7-5p and up-regulating OGT, functional rescue experiments were performed. To begin with, qRT-PCR analysis results ensured the transfection efficiency of pcDNA3.1/OGT (Fig. 5a). qRT-PCR analysis also studied the effects on OGT after knocking down RHPN1-AS1. The results showed that knockdown of RHPN1-AS1 could inhibit OGT expression significantly compared with control group, but then the expression level of OGT was recovered by overexpressing OGT (Fig. 5b). Western blot detected that the change tendency of OGT protein level was same as that of OGT mRNA expression (Fig. 5c). CCK-8 showed that cell viability was inhibited after silencing RHPN1-AS1, but recovered again after overexpressing OGT (Fig. 5d). Flow cytometry manifested that knockdown of RHPN1-AS1 could increase apoptotic cells, but this effect was abolished by OGT overexpression (Fig. 5e). Transwell results indicated that OGT enrichment significantly abrogated the anti-migration and anti-invasion role of RHPN1-AS1 knockdown (Fig. 5f, g). Furthermore, western blot assay further validated this phenomenon, with the results showing that OGT overexpression neutralized the suppressing effects on EMT exerted by RHPN1-AS1 knockdown (Fig. 5h). In general, these results indicate that RHPN1-AS1 modulates the expression of OGT via binding with miR-7-5p, thus regulating the development of CRC. To further elucidate the regulatory effects of RHPN1-AS1 in CRC progression in vivo, we inoculated HCT-116 cells transfected sh-Ctrl and sh-RHPN1-AS1#1 into nude mice. Knockdown of RHPN1-AS1 could slow down xenograft tumor growth than control group (Fig. 5i). Tumor volume and weight were also obviously lessened in sh-RHPN1-AS1 group compared with negative control (Fig. 5j, k). These results suggested that RHPN1-AS1 knockdown could suppress CRC xenograft tumor growth in vivo. In a summary, RHPN1-AS1 serves as miR-7-5p sponge to up-regulate the expression of OGT in CRC cells. MiR-7-5p inhibition and OGT enrichment contribute to neutralizing the suppressing role of RHPN1-AS1 depletion in the malignant behaviors of CRC.

## Discussion

Mounting evidence has demonstrated that lncRNAs are closely associated with normal physiological progresses and pathological change [26]. The aberrantly expressed lncRNAs could be applied as prediction biomarkers for many diseases, including cancers [27–29]. In present study, we found that RHPN1-AS1, as a newly discovered lncRNA, was aberrantly up-regulated in CRC cells than non-tumor one. On the one hand, knockdown of RHPN1-AS1 curbed CRC cell proliferation and promoted apoptosis. On the other hand, RHPN1-AS1 knockdown greatly inhibited cell migration and invasion capacities. These results indicated that RHPN1-AS1 played an oncogenic role in the development of CRC in vitro. STAT3 signal transduction pathway has been reported to be an important pathway in CRC [30]. In present study, transcription factor STAT3 was found to be closely related to the abnormal up-regulation of RHPN1-AS1 in CRC. We identified that STAT3 could activated the transcription of RHPN1-AS1 via ChIP and luciferase reporter assays.

The competing endogenous RNAs (ceRNAs) network has revealed that lncRNAs could restore the expression and biological function of mRNAs via competitively binding to shared miRNAs. This lncRNA-miRNA-mRNA regulatory mechanism exerted significant impacts on malignant tumor progression [31]. In this study, we found that miR-7-5p could interact with RHPN1-AS1. Contrary to the expression status of RHPN1-AS1, miR-7-5p was aberrantly down-regulated in CRC cell lines compared with normal colorectal epithelial cell line. Furthermore, miR-7-5p inhibition could abrogate the suppressing effects of RHPN1-AS1 knockdown on CRC cell proliferation, apoptosis, migration and invasion. This indicated that RHPN1-AS1 was a sponge of miR-7-5p in CRC and its oncogenic function was fulfilled by sponging miR-7-5p expression.

MiRNAs can bind to 3′UTR of target gene with micro response elements (MREs) and further inhibit the cellular function of target gene [32]. OGT was identified a target gene of miR-7-5p by multiple means, including qRT-PCR, luciferase reporter and RIP assays. We found that OGT expression down-regulated most apparently by miR-7-5p overexpression compared with other potential combinable mRNAs. It has been found that OGT is significantly upregulate in colon tissues and may play crucial role in colon cancer carcinogenesis and progression [33]. Furthermore, OGT has been found to promote CRC metastasis by negatively regulating miR-101 and increasing EZH2 stability [34]. In this study, OGT was also significantly up-regulated in CRC cell lines compared with normal colon cell line. Silencing OGT inhibited CRC malignant progresses, including cell proliferation, migration and invasion. Besides, silencing OGT facilitated CRC apoptosis. Rescue experiments revealed that OGT overexpression could abrogate the repressing effects of RHPN1-AS1 down-regulation on CRC cell proliferation, migration and invasion. Overall, these findings initially revealed that lncRNA RHPN1-AS1 promoted CRC progression via acting as ceRNA to up-regulating OGT by sponging miR-7-5p.

## Conclusion

In summary, we initially discovered the underlying regulatory role of RHPN1-AS1/miR-7-5p/OGT network in CRC. Additionally, whether RHPN1-AS1 could be applied as a potential therapeutic target in CRC waits to be further explored in future researches.

## Supplementary information

**Additional file 1: Figure S1.** (A) The apoptosis of sh-OGT#1 or sh-Ctrl transfected CRC cells was measured by flow cytometry assay.

## Data Availability

Research data are not shared.

## Abbreviations

ANOVA: analysis of variance
Bio: biotinylated
CCK-8: cell counting kit-8
ceRNA: competing endogenous RNA
ChIP: chromatin immunoprecipitation
CRC: colorectal cancer
EdU: 5-ethynyl-2′-deoxyuridine
EMT: epithelial–mesenchymal transition
ESCC: esophageal squamous cell carcinoma
FBS: fetal bovine serum
FEZF1-AS1: FEZ family zinc finger 1 antisense RNA 1
FISH: fluorescence in situ hybridization
GLUT-1: glucose transporter 1
IL-6: Interleukin-6
lncRNAs: long non-coding RNAs
mRNA: Messenger RNA
miRNA: microRNA
MREs: micro response elements
MUT: mutant
ns: no significance
ncRNAs: non-coding RNAs
NSCLC: non-small cell lung cancer
OGT: *O*-GlcNAcylation transferase
PBS: phosphate buffer saline
PI: Propidium iodide
PMT: promoter
PVDF: polyvinylidene fluoride
qRT-PCR: quantitative real time polymerase chain reaction
RHPN1-AS1: Rhophilin Rho GTPase binding protein 1 antisense RNA 1
RIP: RNA immunoprecipitation
RIPA: radio immunoprecipitation assay
SD: standard deviation
SDS-PAGE: sodium dodecyl sulfate polyacrylamide gel electrophoresis
STAT3: signal transducer and activator of transcription 3
WT: wild-type
XIRP2-AS1: xin actin binding repeat containing 2 antisense RNA 1
