# Supplementary figures and images for: Long noncoding RNA RHPN1-AS1 promotes colorectal cancer progression via targeting miR-7-5p/OGT axis
Source: Cancer Cell Int. 2020 Feb 18;20:54. doi: 10.1186/s12935-020-1110-9 (PMC7029493; doi:10.1186/s12935-020-1110-9)

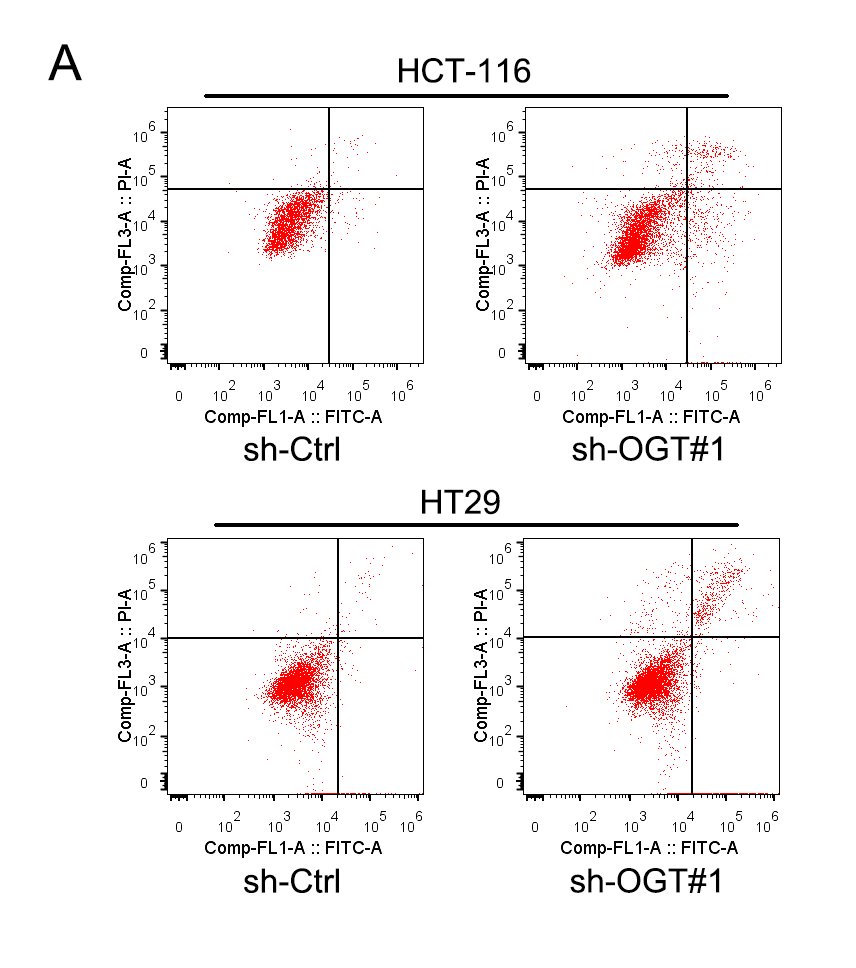

Supplement: Supplementary file 1 — Additional file 1: Figure S1. (A) The apoptosis of sh-OGT#1 or sh-Ctrl transfected CRC cells was measured by flow cytometry assay. [file 12935_2020_1110_MOESM1_ESM.tif]
